# Supplementary material for: Evolution of Patterns of Care and Outcomes in the Real-Life Setting for Patients with Metastatic GIST Treated in Three French Expert Centers over Three Decades
Source: Cancers (Basel). 2023 Aug 28;15(17):4306. doi: 10.3390/cancers15174306 (PMC10487208; doi:10.3390/cancers15174306)
Supplement: Supplementary file 1 [file cancers-15-04306-s001.zip › cancers-2559752-supplementary.pdf]

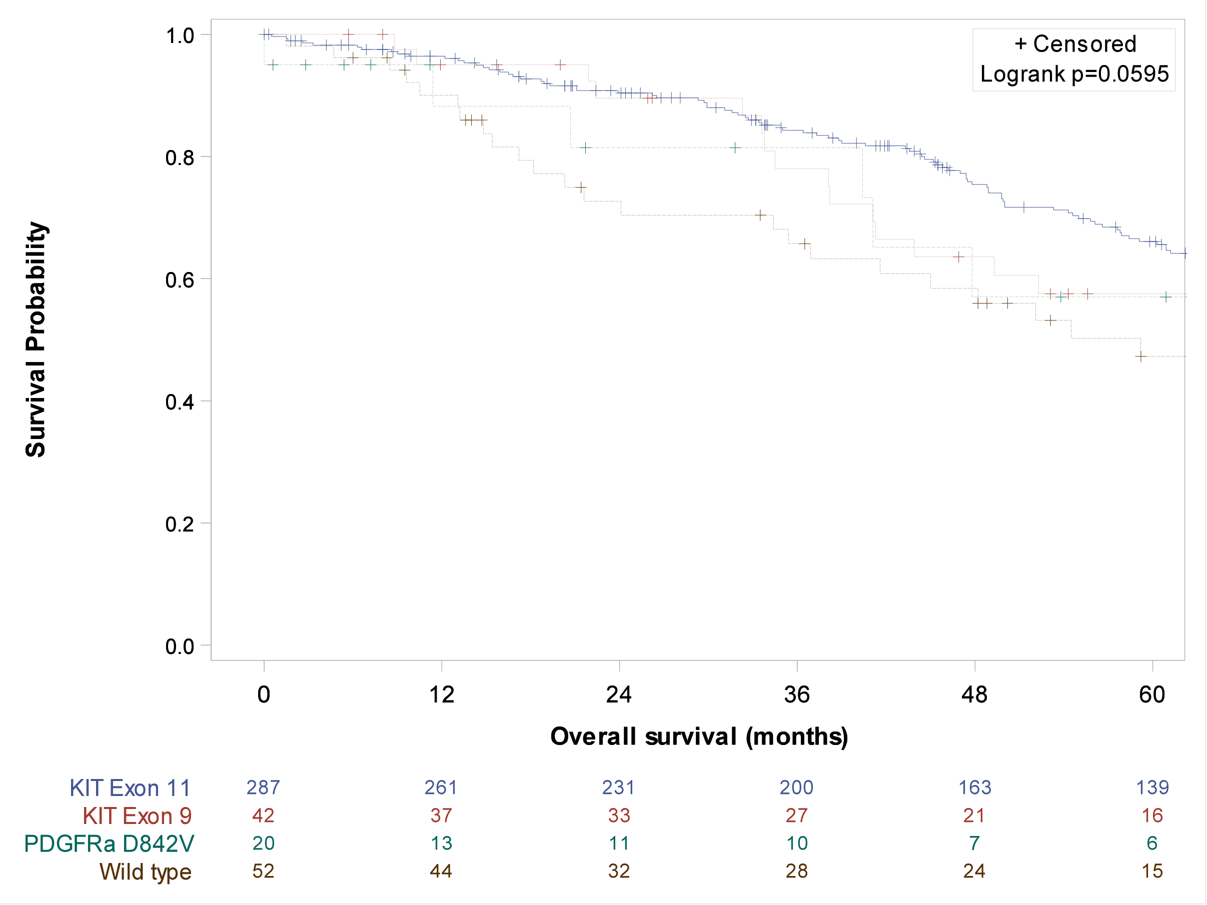

Supplementary Figure S1. Kaplan-Meier curve of overall survival in the metastatic setting according to main molecular subgroups (N = 401).

Median OS in the metastatic setting in the KIT exon 11 mutation subgroup (n = 287), the KIT Exon 9 subgroup (n =42), the PDGFRa D842V subgroup (n= 20) and the wild type subgroup (n=52).

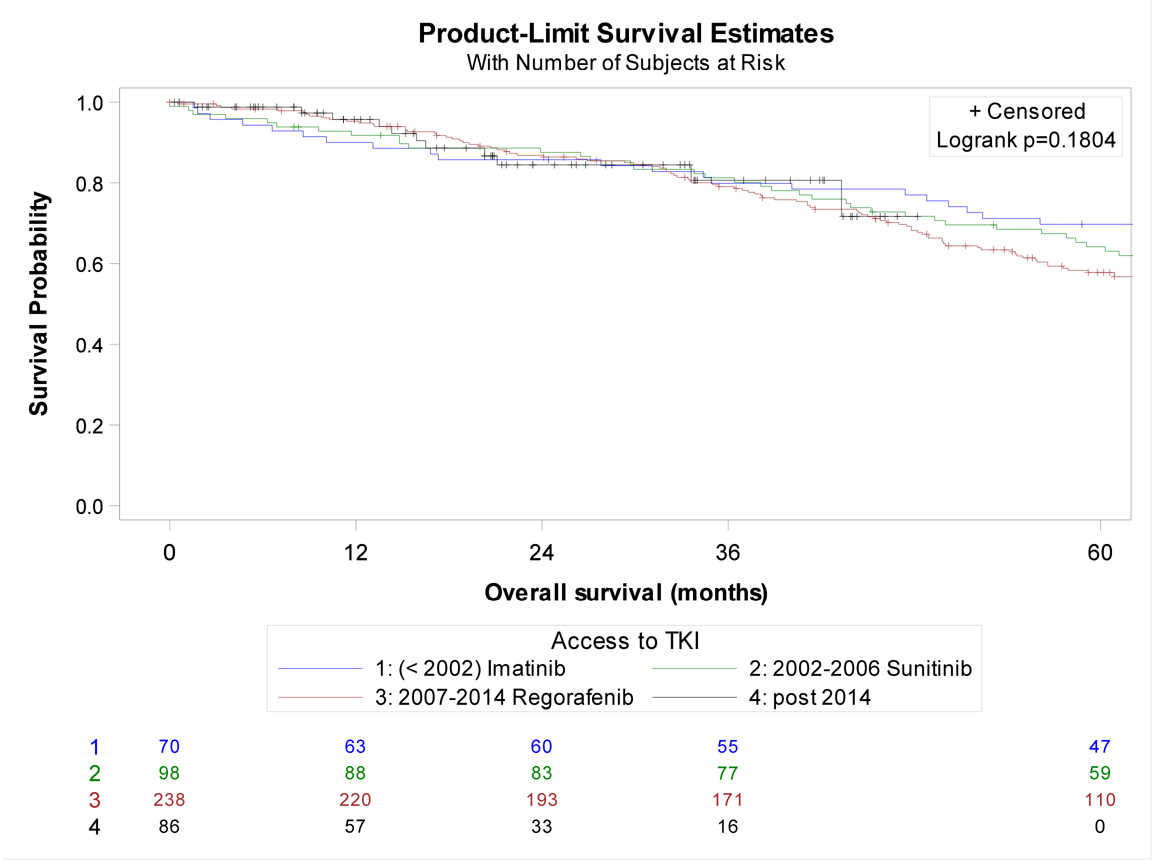

Period of access to TKI	Median OS [95% CI] (months)
< 2002 pre-imatinib approval	101.0 [78.2; 144.2]
2002-2006 pre-sunitinib approval	94.1 [66.5; 122.0]
2007-2014 pre-regorafenib approval	75.5 [60.9; 88.7]
Post 2014 post-regorafenib approval	Not reached

Supplementary Figure S2. Kaplan-Meier curve of overall survival of patients with metastatic GIST treated in three French expert centers according to therapeutic periods with different access to TKI (n = 492)

Supplementary Table S1. Description of baseline characteristics of patients who received imatinib (n = 384) or another molecule (n = 77) as first-line treatment in the metastatic setting.

	Imatinib (n=384)		Other (n=77)	
	**n**	**%**	**n**	**%**
**Sex**				
Male	226	58.9	49	63.6
Female	158	41.1	28	36.4
**Median age at diagnosis, years (min-max)**	59	(19-93)	55	(25-84)
**Tumour site**				
Stomach	156	40.6	32	41.6
Small intestine	151	39.3	30	39.0
Duodenum	18	4.7	5	6.5
Rectum	19	4.9	4	5.2
Peritoneum	16	4.2	3	3.9
Colon	16	4.2	1	1.3
Oesophagus	8	2.1	2	2.6
**Median tumor size, mm (min-max)**	100	(18-400)	110	(35-300)
**Median mitotic index /50HPF (min-max)**	11	(0-350)	10	(0-200)
**Mutational status**				
KIT Exon 11	233	60.7	43	55.8
KIT Exon 9	39	10.2	2	2.6
PDGFRa Exon 18 D842V	12	3.1	4	5.2
Wild type	38	9.9	8	10.4
Other	21	5.5	8	10.4
NA	41	10.7	12	15.6
**Metastasis**				
Synchronous	212	55.2	32	41.6
Metachronous	172	44.8	45	58.4
**Number of metastatic sites**				
Monosite	295	76.8	56	72.7
Multisite	89	23.2	21	27.3
**Locoregional procedure of metastases**				
Yes	185	48.2	42	54.5

	**Imatinib (n=384)**		**Other (n=77)**	
	**n**	**%**	**n**	**%**
No	199	51.8	35	54.5

Supplementary Table S2. Prognostic factors associated with OS in univariate analysis.

Clinical and molecular factors	Pvalue (khi-2)	Hazard Ratio	HR Lower Conf. Limit	HR Upper Conf. Limit
**Sex**				
Female vs Male	*0.038*	0.76	0.59	0.99
**Age at diagnosis, years**				
> 60 vs < 60	*<.001*	1.59	1.24	2.03
**Tumor site**	0.179			
Colon vs Stomach	0.873	0.94	0.44	2.02
Duodenum vs Stomach	0.027	1.76	1.07	2.91
Oesophagus vs Stomach	0.413	1.46	0.59	3.58
Peritoneum vs Stomach	0.059	1.74	0.98	3.11
Rectum vs Stomach	0.795	0.93	0.52	1.65
Small intestine vs Stomach	0.801	1.04	0.79	1.37
**Tumor size (continuous)**	0.411	1.00	1.00	1.00
**Mitotic Index /50 HPF (continuous)**	*<.001*	1.01	1.00	1.01
**Miettinen AFIP scoring**	0.299			
Intermediate risk vs High risk	0.110	0.73	0.50	1.07
Low and very low risk vs High risk	0.517	0.78	0.37	1.66
NA/Missing vs High risk	0.530	1.13	0.78	1.64
**Mutational status**	0.226			
Exon 18 vs Exon 11	0.707	1.13	0.61	2.09
Exon 9 vs Exon 11	0.025	1.63	1.06	2.50
Other vs Exon 11	0.833	1.07	0.58	1.97
Wild type vs Exon 11	0.088	1.41	0.95	2.09
NA vs Exon 11	0.453	1.15	0.80	1.66
**Metastasis at diagnosis**				
Yes vs No	0.715	0.96	0.75	1.22
**Surgery of tumour (primary event)**				
Yes vs No	*<.001*	0.46	0.33	0.65
**Tumour spillage (primary event)**	0.704			
Yes vs No	0.880	1.03	0.71	1.49
NA vs No	0.432	0.85	0.57	1.27
**Margin R (primary event)**	*0.004*			
R1 vs R0	0.059	1.40	0.99	1.99
R2 vs R0	0.001	2.42	1.43	4.11
NA vs R0	0.349	0.79	0.49	1.29

**Status at referral**				
Relapse vs First event	0.733	1.05	0.81	1.35
**Participated in a clin. trial in palliative care in 1st line**				
Yes vs No	0.159	0.83	0.65	1.07
**Participated at least once in a clin. trial in palliative care**	0.639			
Yes vs No	0.404	1.13	0.85	1.49
Missing vs No	0.506	1.27	0.63	2.53
**Loco-regional treatment of metastasis**				
Yes vs No	*<.001*	0.56	0.44	0.72
**Periods of TKI access**	0.183			
(< 2002) Imatinib vs post 2014	0.534	0.80	0.40	1.60
2002-2006 Sunitinib vs post 2014	0.796	0.92	0.47	1.79
2007-2014 Regorafenib vs post 2014	0.658	1.15	0.61	2.18

NA : not available

Supplementary Table S3. Prognostic factors associated with TNT in first line in univariate analysis.

Clinical and molecular factors	Pvalue (khi-2)	Hazard Ratio	HR Lower Conf. Limit	HR Upper Conf. Limit
**Sex**				
Female vs Male	0.233	0.88	0.71	1.09
**Age at diagnosis, years**				
> 60 vs < 60	*0.037*	1.25	1.01	1.55
**Tumor site**	0.608			
Colon vs Stomach	0.913	0.97	0.52	1.79
Duodenum vs Stomach	0.462	1.20	0.74	1.93
Oesophagus vs Stomach	0.807	0.90	0.40	2.05
Peritoneum vs Stomach	0.068	1.62	0.97	2.72
Rectum vs Stomach	0.575	1.15	0.70	1.88
Small intestine vs Stomach	0.879	0.98	0.78	1.24
**Tumor size (continuous)**	0.636	1.00	1.00	1.00
**Mitotic Index /50HPF (continuous)**	*0.037*	1.00	1.00	1.01
**Miettinen AFIP scoring**	0.120			
Intermediate risk vs High risk	0.431	0.88	0.64	1.21
Low risk/very low vs High risk	0.025	0.47	0.24	0.91
NA vs High risk	0.675	1.07	0.77	1.49
**Mutational status**	*0.005*			
Exon 18 vs Exon 11	0.045	1.62	1.01	2.59
Exon 9 vs Exon 11	0.055	1.45	0.99	2.13
Other vs Exon 11	0.403	1.24	0.75	2.07
Wild type vs Exon 11	0.006	1.62	1.15	2.28
NA vs Exon 11	0.011	1.52	1.10	2.11
**Metastasis at diagnosis**				
Yes vs No	0.916	1.01	0.82	1.25
**Surgery of tumour (primary event)**				
Yes vs No	*<.001*	0.59	0.44	0.79
**Tumour spillage (primary event)**	0.498			
Yes vs No	0.241	1.21	0.88	1.68
NA vs No	0.737	1.06	0.76	1.48
**Margin R (primary event)**	*0.001*			
R1 vs R0	0.237	1.22	0.88	1.68
R2 vs R0	<.001	2.50	1.53	4.10

NA vs R0	0.143	0.73	0.48	1.11
**Status At Referral**				
Relapse vs First event	0.453	1.09	0.87	1.36
**Participated in a clin. trial in palliative care in 1st line**				
Yes vs No	*0.027*	0.78	0.63	0.97
**Participated at least once in a clin. trial in palliative care**				
Yes vs No	*0.010*	1.34	1.07	1.68
**Loco-regional treatment of metastasis**				
Yes vs No	0.002	0.71	0.58	0.88
**Periods of TKI access**	0.217			
(< 2002) Imatinib vs post 2014	0.283	1.26	0.82	1.94
2002-2006 Sunitinib vs post 2014	0.565	0.88	0.58	1.34
2007-2014 Regorafenib vs post 2014	0.868	1.03	0.71	1.51

NA : not available

Supplementary Table S4. Prognostic factors associated with OS in univariate analysis for synchronous metastatic patients (n = 259).

Clinical and molecular factors	Pvalue (khi-2)	Hazard Ratio	HR Lower Conf. Limit	HR Upper Conf. Limit
**Sex**				
Female vs Male	0.178	0.78	0.55	1.12
**Age at diagnosis, years**				
> 60 vs < 60	0.017	1.52	1.08	2.15
**Tumor site**	0.444			
Colon vs Stomach	0.244	1.73	0.69	4.35
Duodenum vs Stomach	0.212	1.66	0.75	3.65
Oesophagus vs Stomach	0.218	2.44	0.59	10.07
Peritoneum vs Stomach	0.104	1.72	0.90	3.30
Rectum vs Stomach	0.977	0.99	0.42	2.30
Small intestine vs Stomach	0.603	1.11	0.75	1.64
**Tumor size (continuous)**	0.296	1.00	1.00	1.00
**Mitotic Index /50 HPF (continuous)**	0.026	1.00	1.00	1.01
**Miettinen AFIP scoring**	0.185			
Intermediate risk vs High risk	0.153	0.67	0.39	1.16
Low and very low risk vs High risk	0.371	0.63	0.23	1.72
NA/Missing vs High risk	0.257	1.30	0.83	2.04
**Mutational status****	0.033			
Exon 18 vs Exon 11	0.106	0.31	0.08	1.28
Exon 9 vs Exon 11	0.022	1.83	1.09	3.06
Other vs Exon 11	0.349	0.51	0.13	2.08
Wild type vs Exon 11	0.247	1.45	0.77	2.74
Na vs Exon 11	0.095	1.50	0.93	2.42
**Surgery of tumour (primary event)**				
Yes vs No	*<.001*	0.46	0.33	0.65
**Tumour spillage (primary event)**	0.704			
Yes vs No	0.880	1.03	0.71	1.49
NA vs No	0.432	0.85	0.57	1.27
**Margin R (primary event)**	*0.004*			
R1 vs R0	0.084	1.62	0.94	2.80

R2 vs R0	<.001	3.10	1.68	5.71
NA vs R0	0.844	1.07	0.53	2.16
**Status at referral**				
Relapse vs First event	0.544	0.86	0.52	1.41
**Participated in a clin. trial in palliative care in 1st line**				
Yes vs No	0.150	0.77	0.53	1.10
**Participated at least once in a clin. trial in palliative care**	0.842			
Yes vs No	0.655	1.09	0.75	1.57
Missing vs No	0.780	0.85	0.26	2.73
**Loco-regional treatment of metastasis**				
Yes vs No	0.006	0.62	0.44	0.87
**Periods of TKI access**	0.615			
(< 2002) Imatinib vs post 2014	0.948	0.97	0.40	2.34
2002-2006 Sunitinib vs post 2014	0.725	1.16	0.50	2.70
2007-2014 Regorafenib vs post 2014	0.502	1.32	0.59	2.94

** The proportionality assumption required for the application of a cox PH model does not hold.

NA : not available
